# Data assimilation on mechanistic models of glucose metabolism predicts glycemic states in adolescents following bariatric surgery

**DOI:** 10.3389/fphys.2022.923704

**Published:** 2022-11-28

**Authors:** Lauren R. Richter, Benjamin I. Albert, Linying Zhang, Anna Ostropolets, Jeffrey L. Zitsman, Ilene Fennoy, David J. Albers, George Hripcsak

**Affiliations:** ^1^ Department of Biomedical Informatics, Columbia University Irving Medical Center, New York, NY, United States; ^2^ Division of Pediatric Surgery, Department of Surgery, Columbia University Irving Medical Center, New York, NY, United States; ^3^ Division of Pediatric Endocrinology, Metabolism, and Diabetes, Department of Pediatrics, Columbia University Irving Medical Center, New York, NY, United States; ^4^ Department of Bioengineering, University of Colorado Anschutz Medical Campus, Aurora, CO, United States; ^5^ Department of Biomedical Informatics, University of Colorado Anschutz Medical Campus, Aurora, CO, United States

**Keywords:** type 2 diabetes, data assimilation, mechanistic models of glucose metabolism, pediatrics, bariatric surgery, machine learning, obesity

## Abstract

Type 2 diabetes mellitus is a complex and under-treated disorder closely intertwined with obesity. Adolescents with severe obesity and type 2 diabetes have a more aggressive disease compared to adults, with a rapid decline in pancreatic β cell function and increased incidence of comorbidities. Given the relative paucity of pharmacotherapies, bariatric surgery has become increasingly used as a therapeutic option. However, subsets of this population have sub-optimal outcomes with either inadequate weight loss or little improvement in disease. Predicting which patients will benefit from surgery is a difficult task and detailed physiological characteristics of patients who do not respond to treatment are generally unknown. Identifying physiological predictors of surgical response therefore has the potential to reveal both novel phenotypes of disease as well as therapeutic targets. We leverage data assimilation paired with mechanistic models of glucose metabolism to estimate pre-operative physiological states of bariatric surgery patients, thereby identifying latent phenotypes of impaired glucose metabolism. Specifically, maximal insulin secretion capacity, σ, and insulin sensitivity, S_I_, differentiate aberrations in glucose metabolism underlying an individual’s disease. Using multivariable logistic regression, we combine clinical data with data assimilation to predict post-operative glycemic outcomes at 12 months. Models using data assimilation sans insulin had comparable performance to models using oral glucose tolerance test glucose and insulin. Our best performing models used data assimilation and had an area under the receiver operating characteristic curve of 0.77 (95% confidence interval 0.7665, 0.7734) and mean average precision of 0.6258 (0.6206, 0.6311). We show that data assimilation extracts knowledge from mechanistic models of glucose metabolism to infer future glycemic states from limited clinical data. This method can provide a pathway to predict long-term, post-surgical glycemic states by estimating the contributions of insulin resistance and limitations of insulin secretion to pre-operative glucose metabolism.

## 1 Introduction

As obesity rates rise in the United States, so too does the prevalence of type 2 diabetes mellitus (T2DM) in children and adolescents (Skelton et al., 2009; Hales et al., 2017h). While a number of pharmacotherapies exist to treat T2DM, there are few options approved for use in younger patients, who typically have more aggressive disease (TODAY Study Group, 2021; American Diabetes Association Professional Practice Committee, 2022). As such, bariatric surgery is increasingly used as a treatment for severe obesity and prevention or reversal of T2DM, despite risk of operative complications (Hsia et al., 2012; Aung et al., 2016; Beamish and Reinehr, 2017; Inge et al., 2018a; Armstrong et al., 2019; Bolling et al., 2019; Karasko, 2019; Khattab and Sperling, 2019; American Diabetes Association Professional Practice Committee Draznin et al., 2022). Many patients benefit with significant, sustained weight loss, improvement in quality of life, and improvement of obesity-related comorbidities (Inge et al., 2016; Rubino et al., 2016; Beamish and Reinehr, 2017; Pedroso et al., 2018; Inge et al., 2019). However, an ill-defined subset of this population have sub-optimal outcomes (Montero et al., 2011; Livhits et al., 2012; Toh et al., 2017; Inge et al., 2018a; Inge et al., 2018b). Predicting which patients are most likely to benefit from surgery and how they will benefit is a current challenge aimed to minimize unnecessary risk during a critical period of growth and development.

The prevalence of impaired glucose metabolism (IGM)—here referring to a heterogenous population with impaired glucose tolerance (IGT), impaired fasting glucose (IFG), prediabetes (preDM), or T2DM— is increasing in pediatric populations, and may be underestimated (Sinha et al., 2002; Lee et al., 2006; Nowicka et al., 2011; Buse et al., 2013; Dabelea et al., 2014). IGM is progressive and often goes undiagnosed until later in disease history. It is associated with insulin resistance (IR), the need for more insulin to achieve physiologic effects, i.e., peripheral glucose uptake and suppression of hepatic glucose production (HGP). In the obese state, IR is almost guaranteed as it is directly related to visceral adiposity (Kahn and Flier, 2000), but the extent to which the pancreas can compensate exists on a spectrum. Insulin sensitivity (S_I_) is a measure of the effectiveness of insulin in promoting glucose uptake. It is reciprocally related to insulin resistance. In this context, T2DM is an example extreme IGM, with significant IR coupled with β cell dysfunction and resultant hyperglycemia (Prentki and Nolan, 2006; American Diabetes Association Professional Practice Committee, 2022). The extent to which function can be rescued after progression to T2DM is modifiable to some degree (Lim et al., 2011; Pajvani and Accili, 2015; Taylor et al., 2019; Richter et al., 2020; Holst and Madsbad, 2021; Bartolomé et al., 2022). Duration of disease negatively impacts the probability of resolution, and lifestyle interventions resulting in weight loss during the early prediabetic phase are more likely to prevent progression (Knowler et al., 2002).

Reversal of preDM and prevention of T2DM are therefore considered to be one of the major benefits of bariatric surgery in this age group for which there are limited other options (Aung et al., 2016; Armstrong et al., 2019; Bolling et al., 2019; Khattab and Sperling, 2019; American Diabetes Association Professional Practice Committee Draznin et al., 2022). Indeed, when compared to pharmacologic or lifestyle interventions, bariatric surgery is overall the most successful intervention with respect to glycemic improvements and sustained weight loss (Schauer et al., 2012; Courcoulas et al., 2014; Mingrone et al., 2015). The specific surgery has clear impact on outcomes. The three most prevalent bariatric surgeries in the U.S. are adjustable gastric banding (AGB), Roux-en-Y gastric bypass (RYGB), and vertical sleeve gastrectomy (VSG). In AGB, an inflatable band is placed around the upper part of the stomach creating a small pouch. In RYGB, the jejunum is directly connected to a remnant small pouch of stomach, thereby bypassing the majority of the stomach and the duodenum. In VSG, the majority of the stomach is removed along the greater curvature, creating a narrow tube or sleeve. While AGB is thought to act through purely restrictive mechanisms, VSG and RYGB restrict food intake and increase malabsorption, alter secretion of gut hormones related to satiety and insulin secretion, e.g., glucagon-like peptide-1 (GLP-1) and ghrelin, and change bile acid composition through the change in macronutrients present in areas of the small intestine (Seeley et al., 2015; Mulla et al., 2018; Akalestou et al., 2022). These differing effects can have profound impact on insulin resistance in particular, and the success of VSG and RYGB in comparison to AGB has led to their increased usage (Mulla et al., 2018). Depending on how remission is defined, meta-analyses have shown that 20–80% of adults will have some degree of improvement in T2DM at medium-to-long term follow up (Yip et al., 2013; Elbahrawy et al., 2018; Tsilingiris et al., 2019; Purnell et al., 2021), although there is suggestion of continued impaired β cell function and overestimation of success (Ramos-Levi et al., 2013a; Laferrère and Pattou, 2018). Smaller prospective studies focusing on adolescents suggest T2DM remission may occur in up to 80–90% of patients and improvement of preDM may occur in 70–80% of patients following Roux-en-Y gastric bypass (RYGB), the most drastic surgery that is recommended for adolescents with respect to metabolic intervention and risk of complications (Inge et al., 2014; Inge et al., 2017; Olbers et al., 2017; Stefater and Inge, 2017). However, small sample sizes and relatively homogenous populations (> 70% non-Hispanic white) limit generalizability of results. Predicting which patients are likely to have remission or partial remission of IGM as a result of surgery and thus not develop T2DM remains a challenging task (Ramos-Levi et al., 2013b; Wang et al., 2015; Tsilingiris et al., 2019).

Given the complexity of medical decision making in adolescent bariatric surgery, accurately assessing benefits vs. risks for an individual is critical for patients and their care teams. Glucose dysregulation and obesity can compound existing surgical risks and increase the chance of complications. Although adolescents have lower complication rates compared to their adult counterparts, they may still experience wound infections, anastomotic strictures, leaks, wound dehiscence, abdominal hernias, dehydration, and venous thromboembolism (Lamoshi et al., 2020). In patients with diabetes, poor wound healing and risk of infection are serious considerations and can directly impact the success of the surgery and need for revision (Keidar, 2011). In the long term, patients also have significant malabsorption post-bariatric surgery, resulting in multiple vitamin and mineral deficiencies requiring lifelong supplementation (Bal et al., 2012; Lamoshi et al., 2020). The consequences of this decreased nutrition can be great in adolescents who are still undergoing their growth spurts and accruing bone during their pubertal years (Lamoshi et al., 2020; Weiner et al., 2020; Ou et al., 2022). Although advancements have been made to minimize risk of post-operative complications, improve wound healing, and optimize post-operative nutrition, bariatric surgery remains an aggressive measure taken to improve a patient’s health (Crossan and Sheer, 2022; Dewberry et al., 2022). Therefore, providing accurate information about a patient’s current diabetic state the probability of improvement with surgery can allow for more informed decision making.

Previous studies using statistical or traditional machine learning techniques to predict T2DM outcomes have generally focused on relatively homogenous adult populations who underwent RYGB, with or without genetic information included in analysis. Because available data are sparse, these approaches are subject to error and are not often validated in pediatric populations (Aminian et al., 2017; Cao et al., 2020; Kam et al., 2020). Varying definitions of what successful glycemic outcomes mean also complicate prediction (Buse et al., 2009; Holst and Madsbad, 2021). Outside of surgery type, other potential predictors include anthropometrics (weight, height, body mass index [BMI]), pre-operative disease severity, use of anti-diabetic medications, and presence of comorbidities (DeMaria et al., 2007; Livhits et al., 2012; Dixon et al., 2013; Robert et al., 2013; Panunzi et al., 2015; Wang et al., 2015; Shen et al., 2019). Associations are also seen with baseline biomarkers such as fasting glucose, insulin, C-peptide, triglycerides (TG), C-reactive protein (CRP), and hemoglobin A1c (HbA1c) levels (Ortega et al., 2012; Courcoulas et al., 2013; Inge et al., 2014; Pedersen et al., 2016; Yan et al., 2017). These features, particularly pre-operative disease severity (as measured by duration of disease, labs, and medications) are more homogenous in adolescents, who frequently have preDM rather than T2DM, and are therefore on fewer medications, if any. Additionally, adolescents tend to have lower HbA1c values at baseline prior to undergoing bariatric surgery compared to their adult counterparts. As such, features that are useful predictors in adult surgical response are not necessarily translatable to adolescents.

To provide more personalized predictions, Pedersen, et al. incorporated genetic and clinical information in an artificial neural network to accurately predict short-term discontinuation of diabetes medications at 30 days (Pedersen et al., 2016). The majority of candidate genetic markers were associated with insulin secretion, glucose clearance, or insulin sensitization. While genetics certainly play a role in T2DM, pre-operative genetic analyses are not currently practical for every patient (Hatoum et al., 2011; Okser et al., 2013; Rouskas et al., 2014).

These prior studies provide evidence that an individual’s underlying physiology has long-term implications for treatment outcomes, but existing methods to approximate these physiological pathways may not be practical for use in adolescents (Lee, 2007; Brown and Yanovski, 2014). Glucose tolerance and insulin resistance are frequently estimated using point or dynamic lab proxies due to the expensive and invasive nature of the gold standard for measurement, the hyperinsulinemic-euglycemic clamp (Muniyappa et al., 2018). Oral glucose tolerance tests (OGTTs) are one such approximation used to screen for and diagnose dysglycemia (Olson et al., 2010; Muniyappa et al., 2018; American Diabetes Association Professional Practice Committee, 2022). After fasting (usually overnight), patients are given a fixed dose of liquid glucose (typically 75 g) after which glucose levels are measured at timed intervals. OGTTs can vary in the types of labs drawn, the frequency of sampling, and the duration of the procedure (Muniyappa et al., 2018). In common clinical practice, glucose is measured over two or three hours. Measurements of insulin and C-peptide are not the standard of care; their assays are relatively costly and lack of standardization makes their interpretation less straightforward (Manley et al., 2007; Little et al., 2008; Miller et al., 2009; Tohidi et al., 2017). As such, these labs are typically only collected in research settings.

Fasting insulin and glucose measurements can be used to calculate indices such as the homeostatic model assessment of insulin resistance (HOMA-IR), which can approximate parameters such as peripheral and hepatic insulin sensitivity ${hepa}_{S_{I}}$ as would be assessed in clamp studies or frequently sampled intravenous GTTs (FSIVGTT), a silver standard (Matthews et al., 1985; Bergman et al., 1987; Yeckel et al., 2004). Interpretation of these indices can vary by patient characteristics (e.g., age, sex, ethnicity, body habitus) (Wallace et al., 2004; Gunczler and Lanes, 2006; Nathan et al., 2007; Shaibi et al., 2011; Gutch et al., 2015; Arslanian et al., 2019; Tagi et al., 2019; Kim et al., 2020). In particular, HOMA-IR may not be sensitive to improvements in insulin sensitivity in adolescents (Shaibi et al., 2011; Bryant et al., 2014).

Several mechanistic models of glucose and insulin metabolism have been empirically developed and validated against clamp or FSIVGTT studies to mathematically describe glucose metabolism. Models such as the meal model (Dalla Man et al., 2007), oral minimal model (Cobelli et al., 2014), and ultradian model (Sturis et al., 1991) incorporate insulin secretion rates and glucose elimination to estimate states over varying timescales. Topp et al. developed a mechanistic model that incorporates more granular β cell dynamics, which was extended by Ha et al. to model the development of type 2 diabetes over time and quantify the extent to which different insults in the system contribute to disease (Topp et al., 2000; Ha et al., 2016; Ha and Sherman, 2020). Represented as a system of ordinary differential equations (ODEs), these models have the potential to allow for patient-level characterization of glucose metabolism.

Extracting clinical knowledge from these models is not straightforward, as both the models themselves and their results are often viewed as too abstract for application in clinical practice. However, within these mechanistic models are clinically meaningful physiologic parameters when applied to the appropriate problems, e.g. assessing insulin sensitivity’s relationship with lipoprotein metabolism (Chung et al., 2022). Furthermore, while clamp studies represent a patient’s physiologic state and glucose excursions at a specific point in time, mechanistic models have the potential to elucidate more long-term physiologic states that are difficult to capture clinically.

Knowledge within mechanistic models of glucose metabolism can be exploited via data assimilation, a family of methods frequently used in meteorology and aerospace science (Evensen, 2009; Law et al., 2015). Data assimilation leverages the underlying information about the system contained in these mechanistic models to update current and past states using filtering and smoothing, updates that in turn provide the ability to forecast future states by running the estimated model forward in time. Various filters exist that allow for parameterization and propagation of state uncertainty for non-linear systems such as glucose homeostasis (Kalman, 1960; Wan et al., 2001; Julier and Uhlmann, 2004). In previous work, we used the ultradian and meal models with an unscented Kalman filter (UKF), a sequential method that propagates uncertainty in non-linear systems, as well as deterministic and stochastic optimization methods using techniques such as Markov chain Monte Carlo (MCMC) to estimate both states and parameters, generating a real-time, personalized forecast from free-living data (Albers et al., 2017; Levine et al., 2017). In the free-living data context where data are sparse, we developed a constrained (Albers et al., 2019) version of the ensemble Kalman filter (EnKF) method (Evensen, 2009) that made state and parameter estimates more robust. We also successfully applied these methods to real-time glucose forecasting in the setting of T2DM with OGTT data (Mulgrave et al., 2020). Whereas in the free-living case the focus was accurate glucose state determination, the OGTT case focused more on parameter estimates as a marker for underlying disease.

**FIGURE 1:**
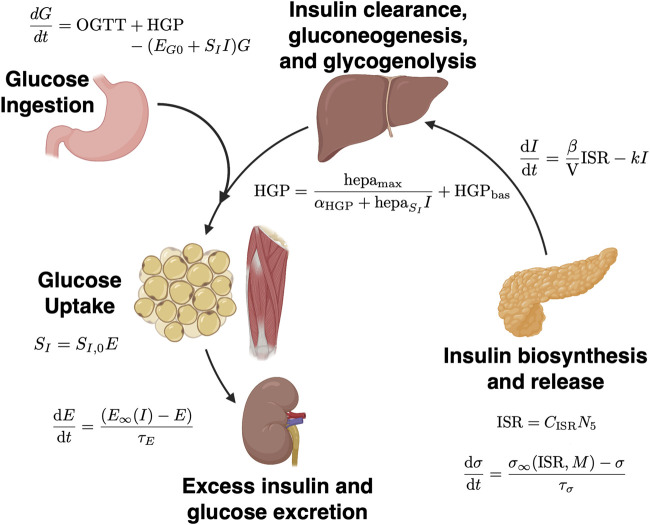
Schematic of mechanistic models of glucose metabolism. Underlying physiologic processes are represented by a series of ordinary differential equations (ODEs). Solutions lie on discrete spaces based on patient physiology at the time of measurements. In data assimilation, after reverse parameter estimation at discrete time points (i.e., insulin secretion capacity, σ, and insulin sensitivity, S_I_), the system state is updated and the ODEs are solved again. Figure adapted from Tokarz et al. (Tokarz et al., 2018) and created with BioRender.com. Relevant equations are outlined in Methods and in Ha and Sherman 2020 (Ha and Sherman, 2020).

Here, we take this prior work to further demonstrate the validity of using physiologic parameters inferred from mechanistic models of glucose metabolism to predict impaired glucose metabolism (IGM) in adolescents at 12-months post-bariatric surgery as compared to other clinical information. We use data assimilation to estimate parameters from an extension of the model initially proposed by Topp et al. incorporating β cell mass (Ha and Sherman, 2020), partially represented in Figure 1. We then train logistic regression models leveraging these parameter estimates derived from data assimilation on a cohort of adolescents who underwent vertical sleeve gastrectomy (VSG) or laparascopic adjustable gastric banding (AGB) at our institution between 2006 and 2020.

## 2 Materials and methods

### 2.1 Extraction of clinical data

Data were collected retrospectively from Columbia University Irving Medical Center (CUIMC) from adolescent patients aged 10–21 who had bariatric surgery between 2006 and 2020. Records were first selected based on the presence of bariatric surgery procedure codes with a diagnosis of obesity on the same day (*n* = 396), a 120-minute OGTT measuring glucose and insulin (at 0, 30, 60, and 120 min) within one year prior to surgery (*n* = 202), a pre-operative HbA1c within 120 days of the OGTT (*n* = 202), and at least one post-operative outcome documented within 6–18 months post-surgery (*n* = 176). These patients were seen through the Center for Adolescent Bariatric Surgery (CABS) at CUIMC. Patients with diganosis codes associated with type 1, cystic fibrosis-related, or gestational diabetes mellitus were excluded. Features as outlined above were extracted for patients who met criteria.

### 2.2 Data pre-processing and manual feature selection

In addition to features necessary to include patients (OGTT glucose and insulin measurements and HbA1c), additional laboratory variables were selected *a priori*. These labs were pre-operative thyroid stimulating hormone (TSH), thyroxine (T4), free T4 (FT4), triiodothyronine (T3), aspartate transaminase (AST), alanine transaminase (ALT), total cholesterol (TC), high-density lipoprotein (HDL), low-density lipoprotein (LDL), triglycerides (TG), and C-peptide. Age was used as a continuous variable. Categorical features included surgery type, demographic data (self-reported race, ethnicity, and sex), and pre-operative presence of specific comorbidities associated with insulin resistance and T2DM: liver disease (including non-alcoholic steatohepatitis [NASH] and non-alcoholic fatty liver disease [NAFLD]), polycystic ovary syndrome (PCOS), dyslipidemia, hypertension (HTN), obstructive sleep apnea (OSA), thyroid disease, metabolic syndrome (MetS), T2DM (by diagnosis code), and abnormal glucose metabolism (by diagnosis code). To be coded as having the comorbidity of interest without manual chart review, at least 25% of all encounters within the appropriate time window had to contain a related diagnosis code (5 years pre-operative; 6–18 months post-operative) (Perotte and Hripcsak, 2013). Features missing in more than 25% of the sample were removed from analysis. The closest labs obtained prior to surgery and the pre-operative OGTT were included as features if there were multiple lab results within the appropriate time window. HOMA-IR was calculated using mass units (Matthews et al., 1985) as follows in Eq. 1:

$${IR}_{HOMA}=\frac{G_{0}\times I_{0}}{405}$$ (1)

where *G*_*0*_ is fasting glucose in mg/dL and *I*_*0*_ is fasting insulin in µIU/mL.

For continuous variables, outliers were defined as those values outside 1.5× the interquartile range (IQR). However, after manual chart review, none of the outliers were removed as none were found to be spurious measurements.

Due to non-normal distributions of the majority of continuous variables, numeric features were scaled and standardized using power transformation with the Box-Cox method (Box and Cox, 1964) prior to hyperparameter tuning. Missing values (*n =* 7, all thyroid function tests) were imputed using iterative imputation using five nearest features with sampling from the prior distribution (Buck, 1960; Buuren and Groothuis-Oudshoorn, 2011; Pedregosa et al., 2011).

In total, we used 21 continuous clinical variables, 23 categorical variables (three multinomial, 11 binary), and six continuous parameter estimates from data assimilation (mean and standard deviations of maximal insulin secretion capacity [σ], insulin sensitivity [S_I_], and their product [σ*S_I_] for an individual’s estimated distributions). Models including HOMA-IR as a feature did not include data assimilation estimates. After data pre-processing, a total of 49 features were included in the most comprehensive model.

### 2.3 Outcome labelling

Patient post-operative outcome classification was coded as a binary variable indicating the presence or absence of impaired glucose metabolism (IGM) at 12 months. To be classified as having IGM, patients could have any one of the following criteria occur in the 6–18-month post-operative window: ≥ 2 elevated HbA1c values, post-operative OGTT G_0_ ≥ 100 mg/dL, post-operative OGTT G_120_ ≥ 140 mg/dL, anti-diabetic drug use (including metformin), or presence of diagnosis codes for T2DM or abnormal glucose metabolism in ≥ 25% of encounters in the post-op window (American Diabetes Association Professional Practice Committee, 2022). If multiple specimens for the same lab were collected within the 6-18-month window, the latest labs were used. Outcome definitions are shown in Table 1 **.**

**TABLE 1:** Outcome definitions for classification as having impaired glucose metabolism (IGM) or normal glucose metabolism (NGM) at 12 months post-bariatric surgery.

	Normal glucose metabolism (NGM)	Impaired glucose metabolism (IGM)	
		Prediabetes	Type 2 diabetes mellitus
HbA1c (%)	< 5.7	5.7–6.4	≥ 6.5
G_0_ (mg/dL)	< 100	100–125	≥ 126
G_120_ (mg/dL)	< 140	140–199	≥ 200
Anti-diabetic medications	None	Metformin or GLP-1a	All other drug classes
Diagnosis codes in encounters within 6–18 months post-op	None or < 25%	Abnormal Glucose in ≥ 25% of encounters	T2DM in ≥ 25% of encounters

G, glucose; GLP-1a, Glucagon-Like Peptide-1 agonist; OGTT, oral glucose tolerance test; OSA, obstructive sleep apnea; T2DM, type 2 diabetes mellitus.

### 2.4 Predictive model training and evaluation

Regularized logistic regression models were trained to predict impaired glucose metabolism (IGM) as a binary outcome on varied subsets of the features as input. The data were first split into 70–30 train-test sets. Hyperparameters were tuned on the train set with stratified nested *k*-fold cross-validation. Tuned hyperparameters included regularization method (*L*2 vs. *L*1), regularization constant (λ = α*n), learning rate [*f*(α)], and max iterations. The loss function was binary cross-entropy loss with balanced class weight. The optimizer was stochastic gradient descent with an adaptive learning rate. To ensure model robustness to random data splitting, we performed 30 independent train-test splits and the results were averaged. The hyperparameters associated with the minimum loss in training were selected for model evaluation.

Because our dataset was imbalanced with respect to outcomes, we chose class weights in the logistic regression models that were inversely proportional to the class frequency. To evaluate the performance of the prediction models, we computed area under the Receiver Operating Characteristic curve (AUROC), precision, recall, and average precision (AP) on out-of-bag estimates from 1,000 bootstrapped samples (*n* = 176)*.* Average precision refers to the weighted mean of precision with respect to recall at all probability thresholds for classification and focuses on how well the models predict the positive class (in our case, post-operative IGM). It is analogous to the area under the precision-recall curve (AUPRC) and better suited for imbalanced datasets compared to accuracy. Baseline performance of a naïve classifier would have an AUPRC equal to the proportion of the positive class in the data (here, 0.318).

### 2.5 Mechanistic glucose metabolism modeling

Relevant equations used in our study are briefly outlined below, with a full description of the model available in the supplemental materials of Ha and Sherman 2020 (Ha and Sherman, 2020; Sherman, 2022).

Eq. 2 describes change in glucose over time as a function of the glucose flux during the OGTT (OGTT), hepatic glucose production (HGP), insulin sensitivity (S_I_), the insulin-independent effectiveness of glucose (*E*_*G*0_), current glucose (*G*), and insulin (*I*). It is given as:

$$\frac{dG}{dt}=\text{OGTT}+\text{HGP}-\left(E_{G0}+S_{I}I\right)G$$ (2)

where HGP is a decreasing function of *I* and hepatic insulin sensitivity (hepa_S_I__) and *E*_*G*0_ represents insulin-independent glucose uptake by peripheral tissues, here fixed at 0.0118 min^−1^.

Eqs. 3, 4 describe change in insulin over time as a function of β cell mass (β), volume of distribution (*V*), insulin secretion rate (ISR), and insulin (*I*). ISR is a function of calcium ion concentration in the β cell cytosol (*C*_ISR_) and the number of primed insulin vesicles at the β cell membrane (*N*_5_). They are given as:

$$\frac{dI}{dt}=\frac{\beta }{V}\text{ISR}-kI$$ (3)

$$\text{ISR}=C_{\text{ISR}}N_{5}$$ (4)

where *k* is a rate constant of insulin clearance. The precise form of *C*_ISR_ as a function of glucose (*G*) and ATP-dependent potassium ion channel (K^+^-ATP) density (γ) can be determined by expanding the equations derived from a slight modification of the steady state of the previously published exocytosis model (Chen et al., 2008).

Eq. 5 shows the change in γ, the density of the β cell membrane ATP-dependent potassium ion channel (K^+^-ATP), as an increasing function of glucose (*G*) on a scale of hours to days. This describes the shift in glucose-dependent insulin secretion in the setting of chronic hyperglycemia, where increases in channel density lead to decreases in insulin secretion. Because of the short time scale of the OGTT, γ was fixed at –0.076 as in prior work (Sherman, 2022) and this equation was not explicitly solved in our methods. It is provided here for clarity on how chronic hyperglycemia affects β cell structure and is given as:

$$\frac{d\gamma }{dt}=\frac{\gamma_{\infty }\left(G\right)-\gamma }{\tau_{\gamma }}$$ (5)

where γ_∞_(*G*) is an increasing sigmoidal function of glucose and τ_γ_ is a time constant.

From the insulin exocytosis model, Eqs. 6, 7 describe the change in the number of vesicles in the β cell granule-membrane complex during docking and priming (*N*_*6*_ and *N*_*5*_, respectively) as functions of K^+^-ATP channel density (γ), glucose (*G*), maximal insulin secretion capacity (σ), and the baseline insulin vesicle priming rate, *r*_2_^0^ (Chen et al., 2008). These equations are given as:

$$\frac{dN_{5}}{dt}=C_{5,5}N_{5}+C_{5,6}N_{6}$$ (6)

$$\frac{dN_{6}}{dt}=C_{6,0}+C_{6,5}N_{5}+C_{6,6}N_{6}$$ (7)

where *C*_*i,j*_ represents the cytosol calcium ion concentration at a given state in exocytosis: *C*_5,5_ is a function of *G* and γ; *C*_*5,6*_ and *C*_6,6_ are functions of *G*, γ, and *r*_2_^0^; C_6,0_ is a function of *G*, γ, and σ, and; *C*_6,5_ is a constant.

Eq. 8 describes the change in the maximal insulin secretion capacity, σ, to compensate for chronic hyperglycemia on a scale of hours to days. It is a unitless scale factor. During the OGTT, σ is assumed to be at steady state. σ is a function of insulin secretion rate (ISR) and β cell metabolism (*M*), where σ increases with increases in ISR and decreases as *M* increases.

$$\frac{d\sigma }{dt}=\frac{\sigma_{\infty }\left(ISR,M\right)-\sigma }{\tau_{\sigma }}$$ (8)

Parameters and non-estimated initial states were set according to their values in previous research (Ha and Sherman, 2020; Sherman, 2022). In experiments where measured insulin values were not included in the estimation optimization, I_0_ was set as 5.63 µIU/mL. The parameters estimated without using a patient's insulin values are denoted with an, $\bar{\sigma S_{I}}$ and $\bar{\sigma^{*}S_{I}}$ .

### 2.6 Estimating parameters using data assimilation

In our methods, we estimated two parameters via data assimilation, insulin sensitivity, S_I_, and maximal insulin secretion capacity, σ. Ordinary differential equations were solved using a Rosenbrock-*W* method (Rosenbrock23) (Rackauckas and Nie, 2017) within the bounds of [0.005, 3] for S_I_ and [0.01, 10] for σ. The posterior distributions of the parameters and the product σ*S_I_ were estimated based on a 500,000 iteration Random Walk Metropolis-Hastings MCMC (Hastings, 1970) chain (excluding a burn-in of 50,000 iterations), assuming a normal distribution error model and uniform priors. A decrease in autocorrelation approaching 0 was appreciated with increasing lags (*k*) near *k* = 500 to support chain convergence, and the number of iterations and burn-in were selected to be orders of magnitude larger than *k* (Roy, 2019). Acceptance rate varied with each patient between 0.2–0.5. Multiple independent chains for a subset of 10 patients provided confidence in the reproducibility of the estimates. The means and standard deviations of the parameters were calculated from the remaining 450,000 iterations (Ge et al., 2018) in an individual’s chain, and these summary statistics were included in logistic regression models.

### 2.7 Descriptive statistics

The majority of continuous features were not normally distributed, so non-parametric methods were used to calculate descriptive statistics on non-transformed data. These data are reported as medians with 95% confidence intervals around the median. The Mann-Whitney-U tests were used to compare continuous variables under these circumstances. χ^2^ or Fisher’s exact tests were used to compare categorical variables depending on the frequencies of the categories. Two-tailed Student’s *t*-tests were used to compare means and 95% confidence intervals of bootstrapped coefficient estimates and scoring metrics.

## 3 Results

### 3.1 Description of data

Out of 396 adolescents who underwent bariatric surgery, 248 had pre-operative OGTTs. Of 202 patients without any missing values in their pre-operative OGTT glucose and insulin measurements, 176 had follow-up within the appropriate time window and were further analyzed. All potential classification methods for impaired glucose metabolism (IGM) demonstrated class imbalance, with ∼35% (*n* = 56) of patients with meeting post-operative criteria for IGM as outlined above. Pre-operative characteristics by group are shown in Tables 2, 3. Using the same criteria to classify IGM post-operatively, 101 of the 176 (57.4%) met criteria for IGM pre-operatively. At baseline, patients who had higher probabilities of having IGM post-operatively were more insulin resistant as measured by HOMA-IR, had higher G_30_, G_60_, and G_120_ values, and had higher I_120_ values.

**TABLE 2:** Pre-operative demographic comparisons by binary post-operative outcome at 12 months. For continuous variables, medians with their 95% CI are shown. Categorical variables are shown as a percentage and count. Significant *p*-values for Mann-Whitney-U tests after Bonferroni correction are in bold (*p* < 0.001).

Variable Name	− IGM *n* = 120	+ IGM *n* = 56	*p*-value
**Pre-operative IGM status** ^**#**^	**45 (54)**	**83.9 (47)**	**< 0.0001**
Operation Age (years)	16.9 (16.6, 17.1)	16.8 (16.5, 17.5)	0.3034
Pre-operative BMI (kg/m^2^)	45.93 (44.66, 47.92)	48.25 (46.20, 51.24)	0.0712
Male (%)	27.5 (33)	33.9 (19)	0.4880
**Race**			
Asian	0 (0)	3.6 (2)	0.1000
Black	17.5 (21)	28.6 (16)	0.1390
Native American	0 (0)	1.8 (1)	0.3180
Other	2.5 (3)	7.1 (4)	0.2110
Pacific Islander	0.8 (1)	0 (0)	1.0000
White	58.3 (70)	46.4 (26)	0.1890
Unknown	20.8 (25)	16.1 (9)	0.5890
**Ethnicity**			
Hispanic	42.5 (51)	51.8 (29)	0.3220
Non-Hispanic	53.3 (64)	51.8 (29)	0.9760
Declined	1.7 (2)	1.8 (1)	1.0000
Unknown	9.2 (11)	1.8 (1)	0.1060
**Surgery Type**			
Roux-en-Y (RYGB)	0.8 (1)	0 (0)	1.0000
Gastric Band (AGB)	42.5 (51)	60.7 (34)	0.0370
Sleeve Gastrectomy (VSG)	55.8 (67)	39.3 (22)	0.0600
Other^x^	0.8 (1)	0 (0)	1.0000
**ICD Codes**			
Abnormal Glucose°	13.3 (16)	33.9 (19)	0.0030
T2DM	8.3 (10)	23.2 (13)	0.0130
Dyslipidemia	29.2 (35)	35.7 (20)	0.4850
GERD	13.3 (16)	14.3 (8)	1.0000
Hypertension	21.7 (26)	41.1 (23)	0.0130
Liver Disease^+^	17.5 (21)	21.4 (12)	0.6780
Metabolic Syndrome	29.2 (35)	32.1 (18)	0.8220
OSA	35 (42)	42.9 (24)	0.4030
PCOS (F, *n* = 87, 37)	19.5 (17)	48.6 (18)	0.0021
Thyroid Disease	4.2 (5)	8.9 (5)	0.2930

^#^ Patients with pre-operative IGM had any one of the IGM definitions pre-operatively. This variable was not used in training any of the models.

^x^Other surgeries refers to non-specific procedure codes for restrictive bariatric surgery.

^+^Liver disease includes non-alcoholic fatty liver disease, non-alcoholic steatohepatitis, and unspecified chronic liver disease.

°Abnormal glucose diagnosis codes exclude any kind of diabetes mellitus.

Abbreviations: BMI, body mass index; CI, confidence interval; GERD, gastroesophageal reflux disease; IGM, impaired glucose metabolism; OSA, obstructive sleep apnea; PCOS, polycystic ovary syndrome; T2DM, type 2 diabetes mellitus.

**TABLE 3:** Pre-operative laboratory value comparisons by binary post-operative outcome at 12 months. Medians with their 95% CI are shown. Significant *p*-values for Mann-Whitney-U tests after Bonferroni correction are in bold (*p* < 0.002).

Variable Name	− NGM *n* = 120	+ IGM *n* = 56	*p*-value
**Baseline HbA1c (%)**	**5.4 (5.3, 5.5)**	**5.8 (5.7, 6.0)**	**< 0.0001**
**Baseline HOMA-IR**	**3.13 (2.76, 3.52)**	**4.53 (3.75, 5.57)**	**1.987** × **10** ^**–3**^
**OGTT Measurements**			
G_0_ (mg/dL)	86.5 (84, 88)	87.5 (86, 93)	0.1081
**G**_**30**_ **(mg/dL)**	**128 (123, 134)**	**140.5 (134, 146)**	**1.312** × **10** ^**–3**^
**G**_**60**_ **(mg/dL)**	**115.5 (113, 121)**	**133.5 (127, 154)**	**2.2** × **10** ^**–4**^
**G**_**120**_ **(mg/dL)**	**98 (93, 104)**	**113.5 (109, 122)**	**1.5** × **10** ^**–4**^
I_0_ (µIU/mL)	14.5 (13.0, 17.0)	20.0 (17.0, 24.0)	4.033 × 10^–3^
I_30_ (µIU/mL)	65.0 (52.0, 81.0)	70.5 (52.0, 87.0)	0.8265
I_60_ (µIU/mL)	63.0 (50.0, 72.0)	62.5 (49.0, 90.0)	0.9456
**I**_**120**_ **(µIU/mL)**	**36.5 (27.0, 50.0)**	**58.5 (47.0, 94.0)**	**1.17** × **10** ^**–4**^
**Other Labs**			
Total Cholesterol (mg/dL)	164 (159, 171)	164 (155, 170)	0.6057
HDL Cholesterol (mg/dL)	42 (41, 44)	43 (41, 47)	0.3700
HDL Cholesterol (mg/dL) (M, *n* = 33, 19)	39 (35, 44)	39 (34, 44)	0.9317
HDL Cholesterol (mg/dL) (F, *n* = 87, 37)	43 (42, 47)	46 (43, 51)	0.2019
LDL Cholesterol (mg/dL)	94 (92, 104)	101 (94, 105)	0.7447
Triglycerides (mg/dL)	103 (93, 113)	87.5 (78, 114)	0.4017
ALT (IU/L)	18.5 (16, 21)	20 (17, 25)	0.3676
AST (IU/L)	19 (18, 19)	18 (18, 21)	0.8286
TSH (µIU/L)	2.3 (2.0, 2.7)	2.8 (2.3, 3.7)	0.4416
Free T4 (ng/dL)	1.14 (1.1, 1.17)	1.16 (1.13, 1.22)	0.2113
Total T4 (µg/dL)	8.2 (8.0, 8.8)	8.7 (8.3, 9.3)	0.3143

Abbreviations: ALT, alanine aminotransferase; AST, aspartate aminotransferase; CI, confidence interval; G, glucose; HbA1c, hemoglobin A1c; HDL, high-density lipoprotein; HOMA-IR, homeostatic model assessment of insulin resistance; I, insulin; IGM, impaired glucose metabolism; LDL, low-density lipoprotein; NGM, normal glucose metabolism; OGTT, oral glucose tolerance test; TSH, thyroid stimulating hormone; T4, thyroxine.

A total of 202 patients met inclusion criteria, but 26 of them did not have follow-up within 6–18 months of surgery. One patient was lost to follow-up due to death within three months of surgery. The proportions of patients lost to follow-up were statistically different between those with pre-operative IGM (Fisher’s exact test *p* = 0.035): nine met two or more pre-operative IGM criteria (three with elevated G_0_, one with elevated G_120_, six with multiple elevated HbA1c values, one on anti-diabetic medication).

### 3.2 Data assimilation results

**FIGURE 2:**
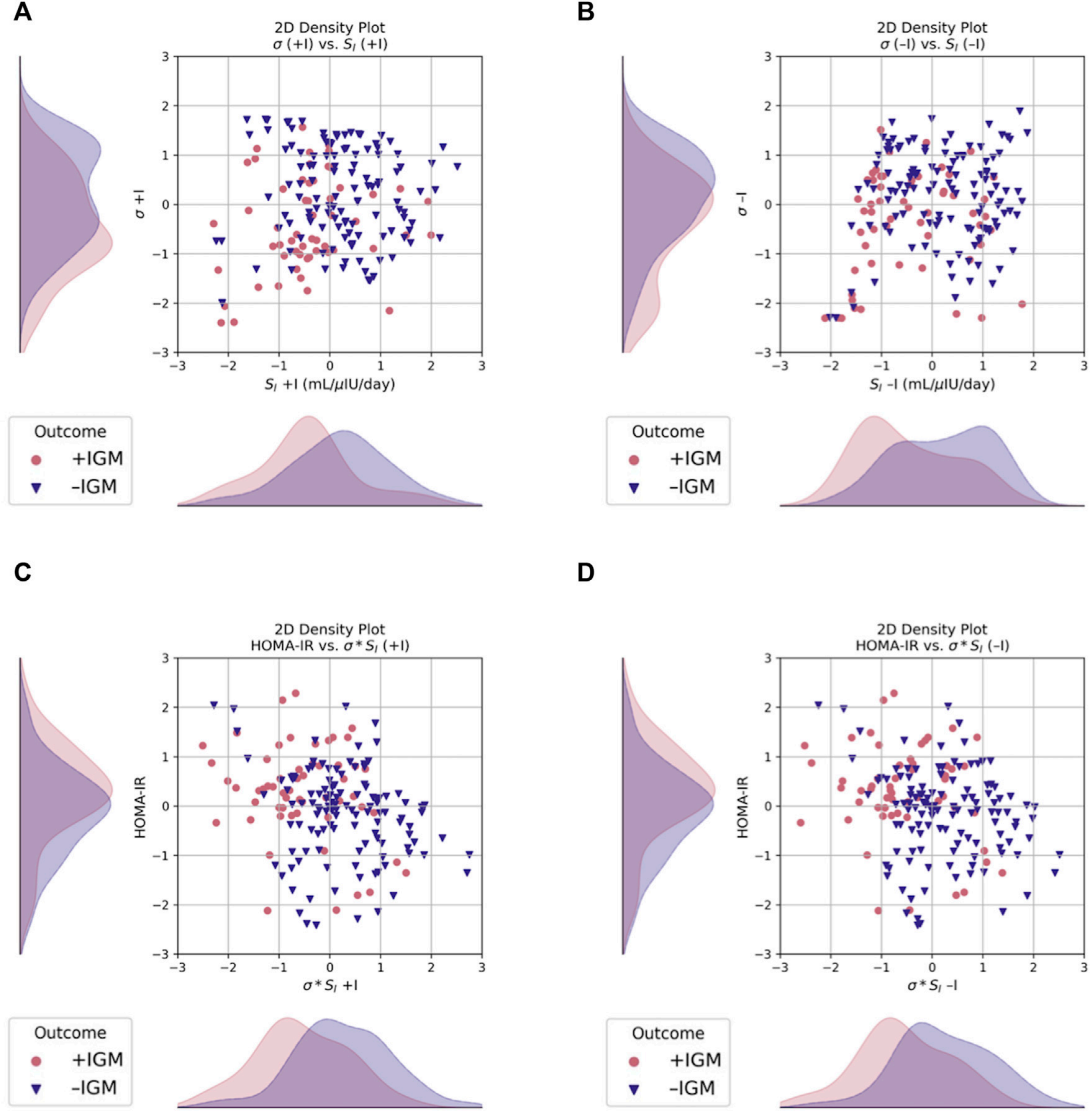
Scatter and 2D kernel density estimation plots, stratified by post-operative outcome at 12 months. Data are shown after Box-Cox transformation for visualization. **(A)** Incorporating measured insulin measurements in the data assimilation estimations increases separation of the estimated probability distributions of σ between groups, as compared to **(B)** without insulin, where S_I_ is more separated but σ has more overlap. **(C)** σ*S_I_ had better separation compared to HOMA-IR when estimated both with and **(D)** without insulin. Blue triangles, normal glucose metabolism (–IGM); red circles, impaired glucose metabolism (+IGM).

Parameter estimates from data assimilation are summarized in Table 4 and the probability distribution of their means across the cohort as estimated by kernel density estimation are shown in Figure 2. The post-operative + IGM group had statistically significantly lower baseline insulin sensitivity (S_I_) and maximal insulin secretion capacity (σ) values compared to the post-operative − IGM group, irrespective of inclusion of insulin in the data assimilation.

**TABLE 4:** Data assimilation-derived mechanistic model parameter estimate comparisons by binary post-operative outcome at 12 months. The parameters S_I_, σ, and σ^*^S_I_ refer to the means of the posterior distributions for each patient. Overlined parameters were estimated without insulin values. For comparisons, medians and 95% confidence intervals of the median are shown. All comparisons had significant *p*-values for Mann-Whitney-U test after Bonferroni correction (*p* < 0.008).

**Parameter Name**	**– IGM *n* = 120**	**+ IGM *n* = 56**	***p*-value**
$\bar{S_{I}}$	0.403 (0.249, 0.571)	0.086 (0.054, 0.207)	1.70 x 10^-5^
$S_{I}$	0.356 (0.284, 0.420)	0.155 (0.129, 0.178)	< 0.0001
$\bar{\sigma^{*}S_{I}}$	0.947 (0.651, 1.539)	0.207 (0.157, 0.379)	< 0.0001
$\sigma^{*}S_{I}$	0.908 (0.763, 1.510)	0.255 (0.146, 0.547)	< 0.0001
$\bar{\sigma }$	4.715 (4.414, 4.976)	4.083 (3.582, 4.399)	0.0011
σ	3.424 (2.627, 4.630)	1.596 (1.195, 3.169)	0.0003

Abbreviations: IGM, impaired glucose metabolism; σ , maximal insulin secretion capacity; $S_{I}$ , insulin sensitivity.

The marginal posterior densities of the parameters were estimated for each individual patient using MCMC. The marginal posterior density of S_I_ was sharply peaked away from its bounds on manual inspection for a random subset of patients, giving us confidence in its mean estimator. The density for σ*S_I_ was also sharply peaked away from its bounds (not shown).

### 3.3 Prediction model results

For the most complex model, data assimilation estimates were negatively correlated with the probability of having post-operative impaired glucose metabolism (IGM) at 12 months. Across models that contained them, HbA1c and I_120_ were positively correlated with the probability of having post-operative IGM at 12 months. However, the model coefficients were overall not significantly different from zero for all models (not shown). When ranked by magnitude, the largest coefficients, when included, were coefficients for HbA1c, I_120_, and G_60_.

Selected AUROC and average precision score comparisons are shown in Tables 5, 6, respectively.

**TABLE 5:** AUROC comparisons between models trained on subsets of features with and without data assimilation. Models are presented in alphabetical order with the better performing model on the left (Model A). Clinical Vars refers to all clinical features in Tables 2, 3 except pre-operative IGM status and HOMA-IR. Clinical Vars^–*Ins*^ refers to the same set of features in Clinical Vars after removing insulin measurements. Overlined parameters were estimated without insulin values. Significant *p*-values for two-tailed Student’s *t*-test after Bonferroni correction are shown in bold (*p* < 0.004).

	**Model A**	**A AUROC Mean (95% CI)**	**Model B**	**B AUROC Mean (95% CI)**	***p*-value**
**1**	**Clinical Vars**	**0.7655 (0.7622, 0.7689)**	**Clinical Vars** ^**–*Ins***^ **+** $\bar{\sigma^{*}S_{I}}$	**0.7511 (0.7475, 0.7547)**	**< 0.0001**
2	Clinical Vars + HOMA-IR	0.7659 (0.7625, 0.7692)	Clinical Vars	0.7655 (0.7622, 0.7689)	0.8942
3	Clinical Vars^–*Ins*^ + $\bar{\sigma^{*}S_{I}}$	0.7511 (0.7475, 0.7547)	Clinical Vars^–*Ins*^	0.7491 (0.7455, 0.7527)	0.4351
4	Clinical Vars + σ + $S_{I}$ + $\sigma^{*}S_{I}$	0.7678 (0.7644, 0.7713)	Clinical Vars	0.7655 (0.7622, 0.7689)	0.3573
5	Clinical Vars + σ + $S_{I}$ + $\sigma^{*}S_{I}$	0.7678 (0.7644, 0.7713)	Clinical Vars + HOMA-IR	0.7659 (0.7625, 0.7692)	0.4282
6	Clinical Vars + $\sigma^{*}S_{I}$	0.7700 (0.7665, 0.7734)	Clinical Vars	0.7655 (0.7622, 0.7689)	0.0728
**7**	Clinical Vars + $\sigma^{*}S_{I}$	0.7700 (0.7665, 0.7734)	Clinical Vars + HOMA-IR	0.7659 (0.7625, 0.7692)	0.0952
**8**	**Glucose + Insulin + HbA1c +** $\sigma^{*}S_{I}$	**0.7627 (0.7594, 0.7659)**	**Glucose + Insulin + HbA1c +** σ **+** $S_{I}$ **+** $\sigma^{*}S_{I}$	**0.7451 (0.7416, 0.7486)**	**< 0.0001**
**9**	**Glucose + Insulin +** σ **+** $S_{I}$ **+** $\sigma^{*}S_{I}$	**0.7463 (0.743, 0.7496)**	**Glucose + Insulin**	**0.7337 (0.7303, 0.7371)**	**< 0.0001**
10	$\bar{\sigma^{*}S_{I}}$	0.7380 (0.7346, 0.7415)	Glucose + Insulin	0.7337 (0.7303, 0.7371)	0.0790
11	$\bar{\sigma^{*}S_{I}}$	0.7380 (0.7346, 0.7415)	Glucose + Insulin + HOMA-IR	0.7317 (0.7283, 0.7350)	0.0089

Abbreviations: CI, confidence interval; HbA1c, hemoglobin A1c; HOMA-IR, homeostatic model assessment of insulin resistance; σ , maximal insulin secretion capacity; $S_{I}$ , insulin sensitivity.

**TABLE 6:** Average precision comparisons between models trained on subsets of features with and without data assimilation. Models are presented in the same order as in Table 5. Clinical Vars refers to all clinical features in Tables 2, 3 except pre-operative IGM status and HOMA-IR. Clinical Vars^–*Ins*^ refers to the same set of features in Clinical Vars after removing insulin measurements. Overlined parameters were estimated without insulin values. Significant *p*-values for two-tailed Student’s *t*-test after Bonferroni correction are shown in bold (*p* < 0.004).

	**Model A**	**A AP Mean (95% CI)**	**Model B**	**B AP Mean (95% CI)**	**p-value**
1	Clinical Vars	0.6200 (0.6148, 0.6252)	Clinical Vars^–*Ins*^ + $\bar{\sigma^{*}S_{I}}$	0.613 (0.6074, 0.6185)	0.0700
2	Clinical Vars + HOMA-IR	0.6209 (0.6155, 0.6262)	Clinical Vars	0.6200 (0.6148, 0.6252)	0.8200
3	Clinical Vars^–*Ins*^ + $\bar{\sigma^{*}S_{I}}$	0.613 (0.6074, 0.6185)	Clinical Vars^–*Ins*^	0.6075 (0.6021, 0.6129)	0.1665
4	Clinical Vars + σ + $S_{I}$ + $\sigma^{*}S_{I}$	0.6253 (0.6197, 0.6308)	Clinical Vars	0.6200 (0.6148, 0.6252)	0.8835
5	Clinical Vars + σ + $S_{I}$ + $\sigma^{*}S_{I}$	0.6253 (0.6197, 0.6308)	Clinical Vars + HOMA-IR	0.6209 (0.6155, 0.6262)	0.2656
6	Clinical Vars + $\sigma^{*}S_{I}$	0.6258 (0.6206, 0.6311)	Clinical Vars	0.6200 (0.6148, 0.6252)	0.1244
**7**	Clinical Vars + $\sigma^{*}S_{I}$	0.6258 (0.6206, 0.6311)	Clinical Vars + HOMA-IR	0.6209 (0.6155, 0.6262)	0.1966
**8**	**Glucose + Insulin + HbA1c +** $\sigma^{*}S_{I}$	**0.6156 (0.6105, 0.6207)**	**Glucose + Insulin + HbA1c +** σ **+** $S_{I}$ **+** $\sigma^{*}S_{I}$	**0.5936 (0.5882, 0.599)**	**< 0.0001**
**9**	**Glucose + Insulin +** σ **+** $S_{I}$ **+** $\sigma^{*}S_{I}$	**0.5841 (0.5791, 0.5892)**	**Glucose + Insulin**	**0.5695 (0.5644, 0.5745)**	**0.0001**
**10**	$\bar{\sigma^{*}S_{I}}$	**0.5990 (0.5939, 0.6041)**	**Glucose + Insulin**	**0.5695 (0.5644, 0.5745)**	**< 0.0001**
**11**	$\bar{\sigma^{*}S_{I}}$	**0.5990 (0.5939, 0.6041)**	**Glucose + Insulin + HOMA-IR**	**0.5662 (0.5611, 0.5713)**	**< 0.0001**

Abbreviations: AP, average precision; CI, confidence interval; HbA1c, hemoglobin A1c; HOMA-IR, homeostatic model assessment of insulin resistance; σ , maximal insulin secretion capacity; $S_{I}$ , insulin sensitivity.

The best performing model used all available clinical variables (*n* features = 43) and σ*S_I_ with an AUROC of 0.77 (95% CI 0.7665, 0.7734) and average precision of 0.6258 (95% CI 0.6206, 0.6311). Our most comprehensive models using all clinical data had similar performances regardless of whether data assimilation estimates or HOMA-IR were included (Tables 5, 6, rows 1–7).

## 4 Discussion

### 4.1 Data assimilation estimates can add clinical information that improves prediction

Our best-performing model used the aforementioned clinical variables combined with the product of maximal insulin secretion capacity and insulin sensitivity, σ*S_I_, with an AUROC of 0.77 (95% CI 0.7665, 0.7734) and average precision of 0.6258 (95% CI 0.6206, 0.6311). This model was nominally better than one using clinical variables alone with an AUROC of 0.7655 (95% CI 0.7622, 0.7689) and average precision of 0.6200 (95% CI 0.6148, 0.6252), but the differences were not significant at *p* = 0.0728 and 0.1244, respectively (Tables 5, 6, row 6).

### 4.2 Data assimilation estimates can infer missing information encoded in insulin measurements

The comparability of our most comprehensive models suggests that the information added by data assimilation is captured in an extensive, but not exhaustive, clinical dataset. Embedded in the electronic health record (EHR) data was a powerful experiment where the effects of bariatric surgery could be thoroughly investigated. Models incorporating insulin, in general, outperformed models that did not include it. However, when insulin measurements are missing, data assimilation can add physiologic information that approaches the predictive ability of the full clinical dataset. For example, when using all other clinical features *except* insulin, the model using the product $\bar{\sigma^{*}S_{I}}$ had nominally improved performance to the model not including any data assimilation estimates, although the *p*-value was not significant (AUROC 0.7511 [0.7475, 0.7547] vs. 0.7491 [0.7455, 0.7527], respectively, *p* = 0.4351; AP 0.6130 [0.6074, 0.6185] vs. 0.6075 [0.6021, 0.6129], *p* = 0.1665) (Tables 5, 6, row 3). While the performance of this same model using $\bar{\sigma^{*}S_{I}}$ performed worse than the model using the full clinical dataset including insulin (AUROC 0.7511 [0.7475, 0.7547] vs. 0.7655 [0.7622, 0.7689], *p* < 0.0001; AP 0.6130 [0.6074, 0.6185] vs. 0.6200 [0.6148, 0.6252], *p* = 0.0700) (Tables 5, 6, row 1), its AUROC was non-inferior at 98.5% with *p* = 0.2326.

Our models further suggest that σ*S_I_, even when estimated sans insulin, can represent the information within an OGTT using glucose and insulin. When compared, our model trained on only $\bar{\sigma^{*}S_{I}}$ had similar or better performance as compared to models trained on the glucose and insulin measurements from an OGTT (AUROC 0.7380 [0.7346, 0.7415] vs. 0.7337 [0.7303, 0.7371], *p* = 0.079; AP 0.5990 [0.5939, 0.6041] vs. 0.5695 [0.5644, 0.5745], *p* < 0.0001) (Tables 5, 6, row 10). The predictive performance of $\bar{\sigma^{*}S_{I}}$ is demonstrated again in comparison with the model incorporating HOMA-IR, which requires a fasting insulin measurement (AUROC 0.7380 [0.7346, 0.7415] vs. 0.7317 [0.7283, 0.7350], *p* = 0.0089; 0.5990 [0.5939, 0.6041] vs. 0.5662 [0.5611, 0.5713], *p* < 0.0001) (Tables 5, 6, row 11).

### 4.3 The mechanistic models were validated using clinical data

The mechanistic models were able to be well estimated, achieving a stable solution with a unique minimum, using our clinical dataset. Furthermore, the mechanistic model output did not contradict nor add unvalidated information, in that the parameters estimated corresponded to variables associated with glucose and insulin metabolism, and not to other clinical variables (e.g., demographics, thyroid function).

### 4.4 Uncertainty of data assimilation estimates results in reduced performance in logistic regression

When included in the model, HbA1c and insulin measures (particularly I_120_) were frequently ranked as the most important predictors by magnitude. The improved prediction ability using A1c or insulin was not replicated by substituting them with data assimilation estimates not using insulin (not shown). While not a complete substitute for the information contained in HbA1c or insulin, when maximal insulin secretion capacity (σ), insulin sensitivity (S_I_), or σ*S_I_ are estimated using insulin measurements, they still improve performance when added to models containing OGTT insulin (AUROC 0.7463 [0.743, 0.7496] vs. 0.7337 [0.7303, 0.7371], *p* < 0.0001; AP 0.5841 [0.5791, 0.5892] vs. 0.5695 [0.5644, 0.5745], *p* = 0.0001) (Tables 5, 6, row 9).

Although in most models the feature coefficients were not statistically different from zero (not shown), the standard deviation of maximal insulin secretion capacity (σ) is given more importance than the actual estimated parameters themselves and HbA1c in the models using all three data assimilation estimates. Furthermore, in the presence of insulin, regularization consistently shrinks the coefficient for insulin sensitivity (S_I_), whereas the coefficient for maximal insulin secretion capacity (σ) increases. The variance in σ's estimation and its overlapping distributions between groups compared to that of S_I_, even when estimated using insulin measurements, likely contributes to the loss of predictive power (Figure 2). Using σ*S_I_ in lieu of σ and S_I_ separately improves model performance (AUROC 0.7627 [0.7594, 0.7659] vs. 0.7451 [0.7416, 0.7486], *p* < 0.0001; AP 0.6156 [0.6105, 0.6207] vs. 0.5936 [0.5882, 0.599], *p* < 0.0001) (Tables 5, 6, row 8).

### 4.5 Disease subtypes can be described by parameter estimates

Differences in pre-operative insulin sensitivity (S_I_) and maximal insulin secretion capacity (σ) estimates were seen between outcome groups when estimated both with and without measured insulin (*p* < 0.05, Figure 2 and Table 4). Regardless of a patient’s pre-operative glycemic status, S_I_ better distinguished those patients who would have post-operative impaired glucose metabolism (IGM) compared to either σ or σ*S_I_ (Figure 2).

Those with post-operative IGM tended to have lower baseline S_I_ and σ values compared to those without it, demonstrating that both may contribute to a patient’s disease, although not necessarily equally. Additionally, those with post-operative IGM had higher pre-operative HbA1c, G_30_, G_60_, and G_120_ and I_120_ values (*p* < 0.05, Table 3). This may reflect defects in second phase insulin secretion, which is associated with decreases in insulin secretion capacity (Ha and Sherman, 2020). Notably, σ*S_I_ and S_I_ values were not correlated with baseline BMI values, demonstrating a seeming disconnect between whole-body adiposity and insulin sensitivity (not shown). Other physiologic parameters that were not estimated using data assimilation in this study, such as insulin secretion rate (ISR) and hepatic insulin sensitivity ( ${hepa}_{S_{I}}$ ), may reveal other disease phenotypes and patient characterizations that could be explored in future work.

### 4.6 Fixed mechanistic model parameters may differ between adolescents and adults

Not all parameters in the mechanistic models can be estimated simultaneously. All models have potential limitations in generalizability beyond the populations studied during initial development. Use of fixed parameters with values derived from clinical studies in adults may not optimally estimate parameters during pubertal states, where hormonal crosstalk greatly influences energy homeostasis. For example, adolescence is marked by a drastic decrease in insulin sensitivity independent of adiposity; euglycemia is achieved by a compensatory and proportional increase in insulin secretion (Hannon et al., 2006). Insulin secretion rates will therefore be elevated in this age group compared to their adult counterparts, and estimated parameter bounds may differ considerably.

Other parameters which may be relatively constant in adulthood might be more dynamic in adolescents. Obesity and age impact β cell mass and proliferation, which were set as fixed components of these mechanistic models (Saisho et al., 2013; Michaliszyn et al., 2014). The fasting value of γ, representing the K^+^-ATP channel density on the β cells, was set to its example value of –0.076 (Sherman, 2022). However, γ plays an important role in regulating β cell physiology and glucose-mediated insulin secretion. Not properly tuning the fasting value of γ may adversely affect the estimates of the parameters regulating β cell physiology such as maximal insulin secretion capacity (σ) and insulin priming rate (*r*_2_^0^). These effects likely reduced the prediction capabilities of our estimates.

Finally, the assumption that estimates based on the 120-min-OGTT approximate parameters as they would be estimated by a hyperinsulinemic euglycemic clamp may be violated in adolescents. Recently, validation of the oral minimal model in adolescents showed that the 120-min-OGTT underestimates insulin sensitivity compared to longer OGTTs, which is not the case for adults (Bartlette et al., 2021). Complicating this suboptimal approximation is the erratic behavior of OGTT measurements, even in euglycemic patients. Glucose and insulin assays can be imprecise, particularly in periods where glucose and insulin are changing rapidly (i.e., after a meal), and removing spurious OGTT measurements can improve performance (Abohtyra et al., 2022).

### 4.7 Binary classification of outcomes obscures a heterogenous population

Because of the relatively rare frequency of overt diabetes pre-operatively and varying sensitivity of common measurements in adolescents, the post-operative glucose metabolism outcome was coded as a binary variable: normal glucose metabolism (NGM) or impaired glucose metabolism (IGM). However, there are likely multiple sub-phenotypes present in these groups, which would negatively impact prediction models.

Though the diagnostic threshold for T2DM is HbA1c ≥ 6.5%, we used HbA1c ≥ 5.7%, the threshold for preDM, to indicate any IGM to improve sensitivity for detecting T2DM in adolescents (Nowicka et al., 2011). The exact interpretation of HbA1c must be considered along with an individual’s hemoglobin concentration and structure (Radin, 2014). In particular, studies have shown that traditional HbA1c thresholds for T2DM (≥ 6.5%) are imperfect for diagnosis in adults and may significantly underestimate the prevalence of T2DM in adolescents (Nowicka et al., 2011; American Diabetes Association Professional Practice Committee Draznin et al., 2022). Furthermore, given the frequency of anemia in bariatric surgery patients, HbA1c may misrepresent average blood glucose levels and thus, normal values are insufficient to exclude IGM phenotypes. As such, HbA1c was used as a feature in a logistic regression rather than as the outcome in a linear regression.

All patients were included for analysis regardless of pre-operative IGM status. Notably, neither hyperinsulinism nor insulin resistance were used to code IGM. As insulin resistance is tightly coupled with visceral adiposity, the likelihood that all patients had insulin resistance as the only manifestation of their IGM phenotype is high; this is supported by the lower S_I_ values and elevated HOMA-IR calculations of patients at baseline (Kahn and Flier, 2000; Stern et al., 2005). Nonetheless, the decision to focus on glucose rather than insulin perturbations in outcome labeling likely impaired the ability of our models to identify those with IGM-like phenotypes post-operatively, particularly as the outcome is not granular enough to distinguish this heterogenous population.

### 4.8 Limitations

#### 4.8.1 Small sample size

Our methods were hindered by a small, imbalanced, and homogenous sample from a single institution. In addition to increasing sample size by using incomplete OGTT data, future work could address class imbalance by under sampling the majority class or removing redundant information. Our sample size relative to features was exacerbated by patient attrition, likely not at random.

#### 4.8.2 Bias in clinical measures

Our cohort is more diverse with respect to ethnicity and race compared to previously reported studies, which may impact the ability of a model to predict outcomes when using estimators such as HbA1c. Race differences in outcomes have been seen in other studies and have been attributed to poor calibration of models or measurements across heterogenous populations (Wallace et al., 2004; Olson et al., 2010; Tharakan et al., 2017). However, race differences between Black and white adolescents have also been noted in hyperinsulinemic-euglycemic clamp studies, so the true difference in T2DM development is still unclear (Michaliszyn et al., 2017).

#### 4.8.3 Complexity and validation of electronic health records

Our study did not occur in the context of a clinical trial and is subject to the constraints of EHR data. The most effective use of EHR data applies knowledge of how data are inputted into the system while also understanding the underlying medical decision making process. Improper automatic encoding of features or outcomes could have negatively impacted our models’ predictive abilities. In the case of our analyses, features such comorbidities and drug information are less reliable than laboratory values, and awareness of missing data is not guaranteed—that is, the EHR datasets are not complete (Weiskopf et al., 2013). For example, formal diagnoses of obesity-related liver diseases (i.e., NAFLD and NASH) use a liver biopsy to confirm pathology, which can be done while a patient is undergoing bariatric surgery. However, the diagnosis codes for *suspected* liver disease may not be reflected in the EHR, and if they are inputted, they are done so irregularly. This was the case in our patient sample, where many of the comorbidities were mentioned within the patient assessment and/or radiology impressions of notes, but not listed as a visit-associated diagnosis code frequently enough to be marked as present by our criteria. Relatedly, medication data are sparse and often inaccurate, with unreliable start and stop dates. Diet information, which is undoubtedly important in this context, is not typically represented at all in structured datasets. Information about mental health and psychiatric comorbidities is intentionally difficult to access for secondary use, and social stigmas surrounding mental health reduce confidence that, absent documentation, no comorbidities are present. Similarly, social determinants of health such as food insecurity, exposure to discrimination, and exposure to adverse childhood events are not captured in this dataset. Compounding these limitations are existing health disparities in access bariatric surgery, leading to selection bias (Tsui et al., 2021).

Manual chart review is the gold standard for extracting clinical information, but this is time intensive and subject to human error. It also cannot account for truly missing information. As this relates to our methods, to avoid misclassifying patients with post-operative IGM, we erred on the side of underestimating the proportion of patients with specific comorbidities by requiring at least 25% of encounters to contain the relevant diagnosis codes and structured documentation of medications. This may have classified patients as not having IGM post-operatively when in fact they did have some impaired metabolism.

#### 4.8.4 Limitations related to the use of mechanistic models

The use of mechanistic models and oridnary differential equations inherently limits the number of parameters that can be estimated concurrently. Combined with the limitations related to our retrospective, observational analysis, we are limited in our ability to verify the estimated values with respect to a patient’s true physiology. There are several physiologic estimates from data assimilation that could be of use clinically, such as pre-hepatic insulin secretion rate (ISR) or hepatic insulin sensitivity ( ${hepa}_{S_{I}}$ ). Both ISR and ${hepa}_{S_{I}}$ are difficult to capture clinically, and estimation using OGTTs is complex (Cauter et al., 1992; Kjems et al., 2000). However, our choices to estimate maximal insulin secretion capacity (σ) and insulin sensitivity (S_I_) limited our ability to estimate other such important parameters. Generally in our results, more information improved predictions and model accuracy varied in a smooth and logical way. While this does not prove that the ISR estimates incorporated into σ are their true values, it demonstrates that these imperfect features have potential utility in improving patient-level predictions related to surgical outcomes.

Related to the limitations of mechanistic models, our predictive models did not incorporate estimates of hepatic insulin sensitivity ( ${hepa}_{S_{I}}$ ) because ${hepa}_{S_{I}}$ was necessarily fixed in our equations to better estimate σ and S_I_. However, mismatch between peripheral and hepatic insulin sensitivity may better describe subgroups of adolescents with IGM compared to peripheral insulin resistance alone, particularly in the setting of physiologic pubertal insulin resistance (Hannon et al., 2006). Future work should incorporate this critical component of glucose-insulin metabolism into descriptions of patient phenotypes.

Also absent in these models are the effects of circulating incretins, i.e., glucagon-like peptide-1 (GLP-1) and glucose-dependent insulinotropic polypeptide or gastric inhibitory polypeptide (GIP). These gut-secreted, insulinotropic hormones are implicated as one potential therapeutic mechanism of action in metabolic surgeries through their actions on insulin secretion and hepatic insulin clearance (Fetner et al., 2005; Hutch and Sandoval, 2017). Supporting this is the general success of novel classes of anti-diabetic drugs leveraging GLP-1 receptor agonists to manage T2DM and obesity in adults and adolescents (Kelly et al., 2020). However, GLP-1 and GIP are not directly represented in the mechanistic models we used, nor is it currently feasible to directly measure their levels in an outpatient clinical setting. Changes in incretins are indirectly reflected in changes in maximal insulin secretion capacity, σ, where increases in GLP-1 lead to increases in σ through the exocytosis model (Ha and Sherman, 2020). If these mechanistic models are employed to predict future physiologic states on a longer time scale, it is critical to include models which incorporate incretin effects to better model post-surgical physiology.

### 4.9 Future directions

Our model could not capture all features to confidently predict post-surgical glycemic states as a dichotomous outcome despite an extensive collection of laboratory data. We do not believe that these estimates should be broadly disseminated to preclude or exclude patients from receiving indicated care. Rather, with future validation, parameters like σ and S_I_ could inform expectations with respect to potential outcomes. Patients intending to have their prediabetic or diabetic states completely reversed should be informed of the possibility that they may not completely resolve with surgery alone. Quantifying that uncertainty may be accomplished using models like the ones described here. By providing more informed consent, we hope that more patients will be able to have meaningful discussions with their care teams to improve long-term surgical outcomes.

Future studies can incorporate using more longitudinal data to see the trends in insulin secretion capacity (σ) and insulin sensitivity (S_I_) in the pre-, peri-, and post-operative periods. Applying more granular outcome definitions on a continuous scale may better capture patients who might improve in the severity of their disease, but not sufficiently to resolve impaired glucose metabolism. Alternatively, unsupervised machine learning methods could be applied on a larger cohort of patients to identify different pre-operative phenotypes.

Investigation into additional features that may improve predictive performance can also inform future work. Focusing on the direct effects of surgery itself may provide more insight into patient outcomes, especially when certain surgeries (e.g., sleeve gastrectomy or RYGB) have larger metabolic impacts as compared to relatively metabolically modest restrictive procedures like gastric banding. Using models that directly incorporate the effects of GLP-1 and its secretion in response to glucose ingestion, such as that developed by De Gaetano et al., (De Gaetano et al., 2013) should be included in future work, as should assessment of the change in GLP-1 secretion patterns post-operatively. Prospective studies with larger sample sizes or measurement of more stable insulin byproducts such as C-peptide during OGTTs could improve model performance both by adding a more specific feature and through improvement of data assimilation estimates. Manual chart review can provide information about pre- and post-operative anthropometrics to better quantify adiposity. Alternatively, methods to estimate fat-free body mass using more readily available clinical data could be explored. Inclusion of other candidate biomarkers associated with glucose and insulin metabolism such as incretins, growth factors, inflammatory markers, and carrier proteins could be added. Variables related to behaviors (including diet), mental health, and social determinants of health should also be included in future studies attempting prediction in this same population.

This research is a starting point for further investigation into the use of mechanistic models and data assimilation applied to clinical problems. In addition to application of similar techniques to clinical problems outside of prediction, research focusing on solving strategies with sparse or irregularly sampled clinical data could provide robust and reliable methods for future studies.

## 5 Conclusion

We demonstrate that data assimilation captures predictive information about glucose metabolism that is not readily apparent from OGTT measurements alone. Further, we validated that our chosen mechanistic model does not add any additional information than it is meant to represent. The clinical variables combined with the product of maximal insulin secretion capacity and insulin sensitivity, σ*S_1_, produced the best-performing model with AUROC = 0.77 and average precision = 0.6258. This model was nominally better than one using clinical variables alone with AUROC = 0.7655, but the difference was not significant at *p* = 0.07. In some cases, using the individual components of insulin secretion capacity (σ) and insulin sensitivity (S_I_) along with their product reduced prediction model performance.

Looking at whether insulin measurement can be replaced by data assimilation, we found that the model using clinical variables with insulin (AUROC = 0.7655) performed better than the models using clinical variables without insulin but combined with $\bar{\sigma^{*}S_{I}}$ (AUROC = 0.7511, *p* < 0.001). We also found, however, that the difference was small and non-inferior at 98.5%, implying that similar performance can be achieved even without insulin measurements.

If we limit our model inputs to OGTT glucose and insulin values, we found adding data assimilation estimates of insulin secretion capacity and insulin sensitivity (σ, S_I_, and σ*S) significantly increased performance (*p* < 0.001). Models using $\bar{\sigma^{*}S_{I}}$ alone, estimated without insulin, performed marginally better than models using OGTT glucose and insulin with respect to AUROC (0.7380 vs. 0.7337, *p* = 0.08) and had significant improvements in average precision (0.5990 vs. 0.5695, *p* < 0.001).

While data assimilation alone does not significantly improve prediction ability compared to a maximal dataset, the separation of parameter distributions may provide insight into how underlying physiologic processes contribute to a patient’s disease. In this adolescent cohort, low insulin sensitivity *and* low maximal insulin secretion capacity distinguish those patients who are less likely to see glycemic benefits from bariatric surgery. While knowing the extent to which defects in glucose-insulin metabolism contribute to disease is not sufficient to confidently predict surgical outcomes, future research can leverage mechanistic models to infer a patient’s physiology even when certain data are absent.

## Data Availability

The original contributions presented in the study are included in the article/supplementary materials, further inquiries can be directed to the corresponding author.
